# Morning blue light treatment improves sleep complaints, symptom severity, and retention of fear extinction memory in post-traumatic stress disorder

**DOI:** 10.3389/fnbeh.2022.886816

**Published:** 2022-09-12

**Authors:** John R. Vanuk, Edward F. Pace-Schott, Ayla Bullock, Simon Esbit, Natalie S. Dailey, William D. S. Killgore

**Affiliations:** ^1^Department of Psychiatry, College of Medicine, University of Arizona, Tucson, AZ, United States; ^2^Massachusetts General Hospital and Harvard Medical School, Charlestown, MA, United States

**Keywords:** blue light therapy, post traumatic stress disorder, sleep, fear conditioning, fear extinction memory, autonomic reactivity, neurobiological reactivity, fMRI

## Abstract

Disrupted sleep is a major feature in numerous clinical disorders and is related to decrements in affective memory processing. The prevalence of sleep disruption in post-traumatic stress disorder (PTSD) is suggested to be a key feature that exacerbates the impaired ability to recall extinction memories during experimental fear conditioning. We hypothesized that an intervention employing blue-wavelength light therapy (BLT) to regulate sleep and stabilize circadian rhythms in patients with PTSD (i.e., via regulated morning exposure) would be associated with PTSD symptom improvement, decreased sleep-related complaints, as well as improved consolidation and retention of extinction memories relative to a fear conditioning/extinction paradigm. Eighty-two individuals with PTSD underwent a well-validated fear conditioning/extinction protocol with subsequent assignment to receive morning BLUE (BLT) or placebo AMBER (ALT) light therapy daily for 30-min over 6-weeks. Participants returned after the intervention for post-treatment extinction recall, comprised of exposure to the previously conditioned stimuli, with the difference in skin conductance response between the “extinguished” and the “never-extinguished” stimuli at follow-up. Participants also viewed previously conditioned stimuli in a novel context during a functional magnetic resonance imaging (fMRI) scan. BLUE light therapy was associated with improvements relative to correlated decreases between PTSD symptoms and sleep-related complaints. Participants receiving BLT also sustained retention of the extinction memory, while those in the placebo amber light treatment group showed impairment, characterized by the restoration of the extinguished fear response after 6-weeks. Participants in the ALT also demonstrated greater reactivity in the left insula when viewing the previously extinguished fear-conditioned stimuli in a novel context. Daily BLUE-wavelength morning light exposure was associated with greater retention of extinction learning in patients with PTSD when compared to ALT, as supported by both autonomic and neurobiological reactivity. We speculate that improved sleep facilitated by a stabilized circadian rhythm, after fear-learning, led to greater consolidation of the fear extinction memory, decreased PTSD symptom presentation, and associated decreases in sleep-related complaints. Prominent exposure treatments for PTSD incorporate principles of fear extinction, and our findings suggest that blue light treatment may facilitate treatment gains by promoting the consolidation of extinction memories via improved sleep.

## Introduction

Adversity is inherent to life, yet individuals have remarkably different affective experiences and behavioral outcomes under conditions potentially assessed as “adverse.” Individuals experience different types/levels of exposure to stressful life events and demonstrate varying capacities for resilience or vulnerability to those adverse circumstances (Masten et al., 1999). The inter-individual capacity for resilience ranges in contexts from the biological, such as fending off an attacking virus, to behavioral, such as the ability to sustain attention during prolonged periods of sleep deprivation, to emotional, such as the ability to recover rapidly from emotionally challenging setbacks or traumatic experiences. Emotional resilience draws upon diverse cognitive and emotional competencies and differs widely across individuals. Affective responses to the same contextual environment/situation are heterogeneous and dependent on an individual’s appraisal of their impending context (Lazarus, 1999). These appraisals rely on the degree that an individual perceives the setting as a challenge or a threat, in conjunction with their ability to cope with the demands specific to the context (Oken et al., 2015). As such, individuals can have vastly different emotional and behavioral outcomes following the experience of objectively similar adverse/traumatic life events.

In recent decades, there has been an increasing awareness concerning the psychological and physical impact that trauma-based experiences can have on an individual. Rates of exposure to a single traumatic event over the life course are estimated as high as 70%, with approximately 30% of individuals experiencing four or more traumatic events during their life (Roberts et al., 2011). A critical factor to highlight, however, is that not all individuals who experience trauma develop the sustained maladaptive psychological and behavioral responses that manifest as clinically significant posttraumatic stress disorder (PTSD). As such, PTSD can be thought of as a disorder related to recovery rather than initial reactivity to a trauma experience. The inter-individual variability in resiliency and potential to successfully engage in empirically based treatments with sustained improvements remains unclear, likely due to the multitude of factors contributing to these capacities (Van Minnen et al., 2002).

Accumulating evidence suggests that the development and maintenance of PTSD is highly dependent on the functioning of the fear neurocircuitry within the brain. Early work suggested that PTSD was associated with a deficit in the ability of the regulatory regions of the brain (i.e., ventromedial prefrontal cortex) to effectively modulate fear responses within the more primitive limbic structures (i.e., amygdala; Rauch et al., 2006; Shin et al., 2006). Functional connectivity between the medial prefrontal cortex (mPFC) and amygdala is critical to decreasing the negative affective salience of memories and is contingent on proper sleep architecture (van der Helm and Walker, 2010; Pace-Schott et al., 2015b). These brain regions also play a crucial role in cardiac vagal control and emotion regulation capacities (Sakaki et al., 2016). The neurovisceral integration model provides further insight into the brain-body relationship by describing the relationship between mPFC function and autonomic control and implications on emotion and health outcomes related to these biological processes (Thayer and Lane, 2000). The insular cortex is another key area in the brain involved in emotional regulation of learning and memory and is particularly important for the interoceptive experience of anxiety (Liberzon and Martis, 2006; Chellappa and Aeschbach, 2021). While not directly associated with the hypothalamic-pituitary-adrenal axis, insula responses are suggested to play a key role in its activity (Fornari et al., 2012; Linnman et al., 2012). Maladaptive HPA function can interrupt critical diurnal cycles, such as the sleep-wake pattern, impacting emotional health and wellbeing (Killgore et al., 2008; Nader et al., 2010). Notably, a growing body of work demonstrates sleep disturbance as one of the most common complaints by individuals suffering from affective disorders and postulated to be a core dimension in the persistence of both depression and post-traumatic stress disorder (PTSD; van der Helm and Walker, 2010; Germain, 2013; Richards et al., 2020).

Exposure to chronic or severe stress primes the neuroendocrine system to prepare the body and brain for survival. The anterior cingulate cortex (ACC), insula, and amygdala are brain regions that functionally adapt to these types of experiences and can become hyper-responsive to any context assessed as a potential threat (Milad and Quirk, 2012; Pace-Schott et al., 2015b; Alexandra Kredlow et al., 2022). Specifically, exaggerated responses by the amygdala during adverse experiences enhance the encoding of emotionally salient memories (Sharot and Yonelinas, 2008). The affective content of these types of memories can lead to subsequent maladaptive changes in behavior such as physiological hyperarousal, intrusive memories, and persistent nightmares that can continue long after the acute effects of their trauma experience have subsided and have pronounced implications on their daily function and, when severe enough, can manifest as post-traumatic stress disorder (PTSD; Yehuda et al., 2015; Shalev et al., 2017; Ressler et al., 2022). Sleep problems are the most prevalent complaint in individuals with PTSD, with self-report rates as high as 90%, and are postulated to be a driving factor in both the persistence and severity of symptom presentations related to the disorder (van der Helm and Walker, 2010; Germain, 2013; Richards et al., 2020). The sleep problems often observed may be attributable to the occurrence of reduced slow-wave sleep, prolonged time spent in stage 1 sleep, as well as a higher density of rapid-eye movements during sleep cycles (Kobayashi et al., 2007). The severity of sleep disturbance is also associated with PTSD symptom severity, and evidence suggests that sleep problems play a mediating role between exposure to stressors and the manifestation of symptoms contributing to PTSD (Gehrman et al., 2013; Wang et al., 2019; Neylan et al., 2021). Restorative sleep offers one of the most potent non-pharmacologic mechanisms for influencing behavior and affect. A critical insight from research using fear conditioning is that fear extinction learning may not generalize to unextinguished but similar stimuli without post-learning sleep (Pace-Schott et al., 2009). Without proper sleep during a critical post-learning temporal window, an individual’s chance for recovery is significantly decreased, making sleep a viable intervention target for PTSD symptom severity (Pace-Schott et al., 2015a).

From a learning theory perspective, PTSD can be characterized by hyper-responsive reactions to stimuli to which an individual has a conditioned fear response (i.e., stimuli related to the traumatic event). Neuroimaging studies suggest that exposure to fear-based stimuli often results in a dampening of activity in the mPFC in conjunction with amygdalar activation that leads to an emotionally salient memory and subsequent conditioned fear response to that memory (Milad and Quirk, 2012; Shalev et al., 2017; Ressler et al., 2022). Fear extinction learning and memory are proposed to be critical mechanisms linking risk factors such as hormones, genotype, cognition, and sleep disturbance with PTSD symptoms and severity (Zuj et al., 2016). Cognitive therapy treatments targeting PTSD, such as prolonged exposure (PE) therapy, leverage continued exposure to fear-based memories in a safe environment to create new memories that compete with and eventually inhibit the trauma memories (Foa et al., 2007). Of interest, studies in both animals and humans demonstrate a positive association between the quality of sleep after a safety-learning experience and the extinction of conditioned fear responses (Fu et al., 2007; Pace-Schott et al., 2015a). Specifically, research demonstrates learning may not generalize to unextinguished but similar stimuli without post-learning sleep (Pace-Schott et al., 2015a). This is a critical insight relative to applications for cognitive-behavioral treatments. Reductions in fear responses fostered through exposure in the therapy room may sustain in that singular environment but these extinction memories are less likely to transfer and generalize to the broader real world environment if adequate restorative sleep is not obtained following treatment (Pace-Schott et al., 2012; Straus et al., 2017). This lack of safety learning generalizability can result in a “vicious cycle” where patients become trapped in the cyclic nature of worsening sleep exacerbating symptom presentation, while those same symptoms contribute to sleep disruption (Walker and van Der Helm, 2009; Goldstein and Walker, 2014; Pace-Schott et al., 2015b; Colvonen et al., 2019).

Because sleep is so critical for retention of extinction memories, it is vital that sleep interventions be incorporated into treatment for PTSD. While there are a number of pharmacologic treatments for sleep issues, there continues to be a need for non-pharmacologic approaches. Continuous positive airway pressure (CPAP) has been shown to significantly enhance improvements in the inhibition and extinguishing of fear based memory for patients with obstructive sleep apnea (OSA), and suggested as an integral consideration in treatment algorithms for patients with co-morbid PTSD and OSA (Reist et al., 2021). Targeted light exposure is an emerging non-pharmacological method of modulating the sleep-wake cycle. While bright light can be effective, recent research suggests that the blue wavelengths of light (446–477 nm) appear to be the active ingredient that is most effective at producing circadian shifts (Lockley et al., 2006). The observed effects are attributable to the presence of photosensitive retinal ganglion cells, which respond to blue light specifically and have a direct connection to a critical brain region involved in melatonin secretion and circadian rhythms: the suprachiasmatic nucleus of the hypothalamus (Lack et al., 2007; Hankins et al., 2008). Blue light exposure has differential effects on the sleep and wake cycle based on the timing of exposure (Terman, 2007). Interestingly, the impact of light exposure is contingent on core body temperature, with an exposure that occurs after the nadir in body temperature at night resulting in a phase advance of the sleep-wake cycle (earlier to rise, earlier to sleep), while exposure before the nadir has an opposite effect and induces a phase delay on the sleep cycle (Bjorvatn and Pallesen, 2009). Short-wavelength light has also been demonstrated to have acute effects on physiology and behavior, such as decreasing HRV, increasing prefrontal brain activation, along with improving alertness and memory (Münch et al., 2012; Sahin and Figueiro, 2013; Alkozei et al., 2016a; Lazzerini Ospri et al., 2017; Fernandez et al., 2018). Our group has demonstrated the combined effects of blue light therapy (BLT) on circadian timing, neurocognitive performance and neural mechanisms in patients recovering from mTBI, which are characterized by sleep related problems post injury (Alkozei et al., 2017a; Raikes et al., 2020; Bajaj et al., 2021; Killgore et al., 2022). The potent influence of sleep disruption on PTSD symptom severity is well established. Multiple studies have investigated sleep-specific interventions within this population, and recent preliminary findings from two small pilot trials suggested that a light treatment in the green wavelengths (∼500 nm; Zalta et al., 2019) and another using broad-spectrum white light showed promise for improving PTSD symptoms (Youngstedt et al., 2021). However, the full utility of morning BLT for improving sleep and reducing symptom severity in PTSD has yet to be formally investigated. Intervening at the biological level may be critical to treatment, as some work demonstrates that improvements in autonomic function measured by HRV were associated with a reduction in PTSD symptoms for individuals who completed biofeedback treatments in conjunction with cognitive therapy, as compared to individuals who only engaged in cognitive therapy (Tan et al., 2011). These results suggest that improvements in symptom severity in PTSD may be facilitated by appropriately timed BLT.

Here, we conducted a comprehensive assessment of the neurobiological, autonomic, and behavioral outcome changes produced by a 6-week intervention of daily morning blue- wavelength light exposure in individuals with clinically significant levels of PTSD. In a randomized, double-blind, placebo-controlled trial, adults with a verified diagnosis of PTSD and concurrent symptom presentation used an LED lightbox each morning for 30-min within the first 2 h after awakening. Each device was fitted with either BLUE (active treatment) or AMBER (control treatment) LEDs. Sleep/wake activity was monitored via online sleep diaries for 1 week before treatment, and throughout the 6-week intervention period. Participants also completed a cognitive assessment battery, resting HRV monitoring, a fear conditioning paradigm, and functional and structural magnetic resonance imaging (MRI) scans on the day before the treatment period and again upon completion of the intervention. We hypothesized that the blue light intervention would lead to improved sleep via circadian phase advancement, a decrease in the severity of PTSD symptoms, increased vagal control of HRV, and sustained fear extinction learning relative to amber placebo light. Further, we hypothesized that these changes would correspond to decreased amygdala, insular, and ACC activity during a post-treatment fear extinction recall task.

## Materials and methods

Study procedures were evaluated and approved by the Institutional Review Board of the University of Arizona College of Medicine and the U.S. Army’s Human Research Protections Office. All participants provided written informed consent prior to participation.

### Participants

Individuals that experienced an index trauma event consistent with a Diagnostic and Statistical Manual - V (DSM-V) diagnosis of PTSD and were currently experiencing symptoms consistent with such a diagnosis were recruited for the present study. Individuals were recruited via advertisements placed within the local Tucson and surrounding metropolitan areas, including posted flyers, radio advertisements, and various internet ad campaigns. Interested participants contacted the investigators and underwent a thorough telephone screening interview. Potentially eligible participants were then invited to attend an in-person visit to determine full eligibility (Visit 1). Eligible participants were between the ages of 18 and 50 years old, right-handed according to the Edinburgh Handedness Inventory (Oldfield, 1971), primary English speakers (i.e., those who began speaking English as their primary language in the home by 3 years of age), and received a diagnosis consistent with PTSD based on the Structured Clinical Interview for DSM-V (SCID-V) during Visit 1 were included. Potential volunteers were excluded for any history of head injury with loss of consciousness for greater than 30 min, or post-traumatic amnesia for > 24 h, major neurological illness (e.g., epilepsy, multiple sclerosis); chronic medical condition (e.g., heart conditions, cystic fibrosis, diabetes, cancer, HIV/AIDS, HEP C, thyroid problems, high blood sugar) or psychiatric condition (e.g., bipolar disorder/manic or hypomanic episodes, personality disorders, schizophrenia/other psychotic disorders, severe OCD or ADHD), an index trauma occurring before the age of 18, an index trauma occurring 10 years or longer prior to participation in the study, ongoing trauma (e.g., currently being in an abusive relationship) or non-qualifying trauma (e.g., index trauma emotional/verbal abuse, children being taken away by the CPS, divorce, natural deaths by age or illness) that would confound interpretation of results. Other exclusionary criteria included abnormal visual acuity that was not correctable by contact lenses (necessary to see stimuli in the magnetic environment of the scanner), IQ estimate less than 70, metal within the body, pregnancy, or other contraindication for MRI procedures, previous formal treatment with light therapy, history of light-induced migraine or epilepsy, medical complications that could elevate the risk of discomfort associated with light-therapy, use of medications that could affect functional neuroimaging results (e.g., beta-blockers, mood stabilizers, atypical antipsychotics, benzodiazepines, hypertension medication, chemotherapy, photosensitive medications etc.), current suicidal intent based on an assessment conducted by a licensed clinical psychologist, currently taking or anticipating the need to take sleep-inducing medications (e.g., zolpidem) or supplements that have known effects on sleep (e.g., melatonin) during the course of the study, reading test score indicative of less than a 6th grade level of reading comprehension, or drug use (marijuana use was not exclusionary). Past drug dependence (other than marijuana) was not exclusionary if individuals had sustained remission (no drug use in the past 12 months).

Prior to undergoing the clinical assessment, all interested individuals were briefed on the study and provided written informed consent. Participants were then evaluated for PTSD severity using the Structured Clinical Interview for DSM-V (SCID) and were required to meet DSM-V criteria for PTSD at a clinical to subclinical severity. A total of 90 participants were enrolled, however, eight participants failed to complete the study, resulting in a final sample of *N* = 82 (blue light treatment—BLT: *n* = 43; amber light treatment—ALT: *n* = 39). Six of these eight participants were removed due to lack of compliance with study procedures based on data collected through the online portal (e.g., daily use of the light device and completion of the online questionnaire) and two of these participants were completing their participation at the outset of the COVID-19 pandemic, so it was not possible to collect all in-person data from those individuals due to institutional COVID mitigation measures in place at that time. Out of the 82 participants who completed the study, 77 met clinically significant criteria for PTSD on the SCID-V, while 5 were just below the threshold and were listed as “sub-clinical” (BLT *n* = 2; ALT *n* = 3). The proportion of clinical to sub-clinical participants between BLT and ALT groups did not differ significantly (χ^2^ = 0.08, *p* = 0.77).

Basic demographic characteristics for the groups **(BLT: *n* = 43; ALT *n* = 39)** are reported in Table 1. The ratio of males to females between the groups did not differ significantly (χ^2^ = 0.01, *p* = 0.94). In addition, groups did not differ significantly on basic demographic variables including age (*t* = –1.1, *p* = 0.27), years of education (*t* = –0.27, *p* = 0.79), or full-scale IQ as measured by the Wechsler Abbreviated Scale of Intelligence – 2nd Edition (WASI-II) (*t* = 1.26, *p* = 0.21). Similarly, the groups did not differ for age at index trauma (*t* = –0.74, *p* = 0.46) or for years since the index trauma (*t* = –0.79, *p* = 0.43).

**TABLE 1:** Baseline demographic characteristics.

*Baseline Demographics*	Blue (active) *n* = 43		Amber (placebo) *n* = 39		
	*M*	*SD*	*M*	*SD*	*p*-value
Age	31.76	8.80	30.26	8.73	0.27
Education Category	4.91	1.61	4.82	1.50	0.79
Full Scale IQ	100.86	11.08	103.31	13.49	0.21
Age at Index Trauma	28.10	8.46	27.14	8.25	0.46
Years since Index Trauma	3.30	2.27	3.03	2.19	0.43
Male/Female (n)	14/29	–	12/27	–	0.94

### General procedure

**FIGURE 1:**
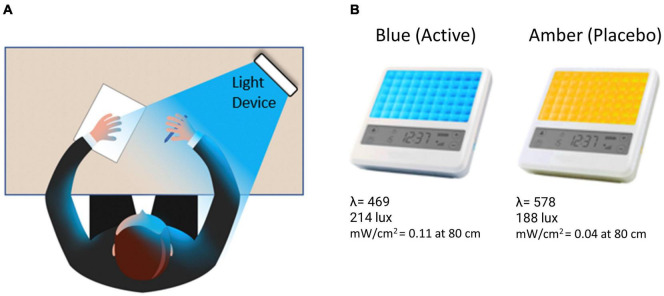
**(A)** The participant was instructed to bathe their face with the light for 30-min each morning by placing the light device at arm’s length on a table at an approximately 45-degree angle. **(B)** Participants received either a blue (active condition) or amber (placebo condition) lightbox fitted with light-emitting diodes.

Over a 7 week period, participants completed three laboratory visits, including two full-day neurocognitive assessments plus neuroimaging scans, and were randomly assigned to complete a 6-week at-home light treatment regimen with either daily BLUE (BLT) or AMBER (ALT) light therapy for 30-min each morning (see Figure 1).

#### Visit 1: Intake

During the first visit, eligible participants completed the informed consent process followed by an evaluation of PTSD severity and other psychopathology using the Structured Clinical Interview for DSM-V (SCID; First et al., 2015). Each eligible participant was shown how to log onto a secure web-based sleep diary to complete daily questions about sleep and activity. Participants were instructed to return to the lab for a baseline neurocognitive assessment and MRI scan 1 week later.

#### Visit 2: Baseline neurocognitive assessment/magnetic resonance imaging scan

After 1 week participants returned for a baseline neurocognitive and neuroimaging assessment, as well as engaged in the initial phase of a fear conditioning paradigm. Participants arrived at the lab and completed a resting electrocardiogram (ECG) assessment, followed by the initial phases of the fear conditioning paradigm (habituation, conditioning, extinction), as well as pre-scan procedures, including a pregnancy test for females. Beginning at 9:00 a.m., participants underwent a neuroimaging scan that included a standard structural T1 magnetization prepared gradient echo (MPRAGE) MRI scan. On a strict, time-controlled schedule throughout the day, participants then completed in-lab sleep, neurocognitive, and psychological assessments. After testing, the participant was provided with a light therapy device with a full demonstration of its use, as well as a printed instruction brochure that provided detailed information about the use of the device.

#### Six-week light therapy

A computer-generated randomization scheme assigned participants to receive either a BLUE or AMBER light device (described in greater detail below) in a double-blind manner (i.e., participants were not informed that there were different colors of lights and all study staff with direct participant contact were blind to the color of the light device assigned). Participants were instructed to use the light device every morning continuously for 30 min, within 2 h of awakening, no later than 11:00 a.m. Participants were instructed to place the lightbox at approximately arm’s length (20–30 in. from their face) and a slight angle (20–40°), so that both sides of the face would be exposed to the light, as well as were encouraged to avoid looking directly at the light diodes to avoid visual discomfort. The device was programmed to turn off automatically after 30 min of continuous use. Participants were also instructed to complete a sleep and light use diary each morning via a secure online portal after finishing light exposure, and also wore wrist actigraphs for the duration of the study (Actiwatch Spectrum Pro^®^, Philips).

#### Visit 3 post-treatment assessment/fear conditioning/magnetic resonance imaging scan

**FIGURE 2:**
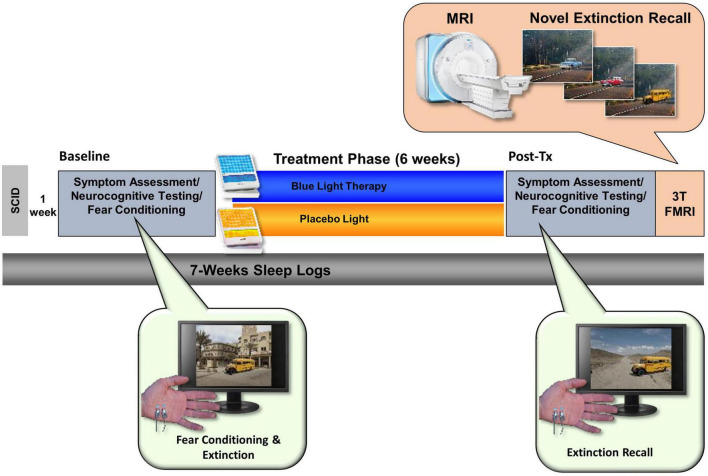
The total study lasted 7 weeks and comprised an initial visit for a structured clinical interview. After 1 week, participants returned for a Baseline assessment day that included neurocognitive testing (including a fear conditioning and extinction protocol). Participants were then randomly assigned to either the Blue Light Treatment (BLT) or placebo Amber Light Treatment (ALT). During treatment, participants used a lightbox (blue or amber) each morning for 30 min over the 6-week period. Participants returned to complete the same assessment battery at the end of the study as well as a 3T fMRI scan while engaging in a novel context extinction recall task.

Upon completion of the 6-weeks of daily morning light exposure, participants returned to the lab for a final assessment session, which was virtually identical in timing and procedures to the baseline session except for an additional Fear Extinction memory component during their MRI scan. At the end of the day, participants returned all equipment and were released from the study (see Figure 2).

### Assessment measures

The following assessment measures and devices were used:

#### Light exposure devices

Participants were provided with either a BLUE or AMBER light therapy device based on randomized assignment (see Figure 1). The devices were manufactured by Philips Electronics (Stamford, CT, United States). All units were identical in design, with the exception of the color wavelength of the LEDs. Each device consisted of a 13.5 × 14 cm plastic-encased table-mounted device with a 10 × 6 array of light emitting diodes (LEDs). For the active BLUE condition, participants were provided with a commercially available Philips goLITE BLU^®^ Energy Light device (Model HF3321/60). The goLITE BLU Energy Light has a narrow bandwidth (peaking at λ = 469 nm, at 214 Lux, and single panel irradiance (mW/cm^2^) = 0.11 at 80 cm). The AMBER devices were designed to be identical to the goLITE BLU devices, with the exception that the LEDs emitted amber light [peaking at λ = 578 nm, at 188 Lux, and panel irradiance (mW/cm^2^) = 0.04 at 80 cm].

#### Post-traumatic stress disorder symptoms

Participants were administered the Structured Clinical Interview for DSM-V (SCID; First et al., 2015) to ensure individuals met diagnostic criteria for a current PTSD diagnosis, as well as the Clinician Administered PTSD Scale for DSM-5 (CAPS-5; Weathers et al., 2018), and Posttraumatic Stress Disorder Checklist for DSM-5 (PCL-5; Blevins et al., 2015), in order to assess PTSD symptom severity at visits two and three.

#### Sleep dysfunction and subjective sleep need assessment

Participants completed the Epworth Sleepiness Scale (ESS) to assess daytime sleepiness (Johns, 1991); the Pittsburg Sleep Quality Index (PSQI), a measure of sleep habits and sleep quality in the past month (Buysse et al., 1991), the Functional Outcomes of Sleep Questionnaire (FOSQ), a measure of the impact of daytime sleepiness on function (Chasens et al., 2009), the Insomnia Severity Index, a measure of both nighttime and daytime insomnia components (Bastien et al., 2001), and the Disturbing Dreams and Nightmares Severity Index (DDNSI), a measure of the frequency and severity of nightmares (Krakow et al., 2002).

#### Fear conditioning

**FIGURE 3:**
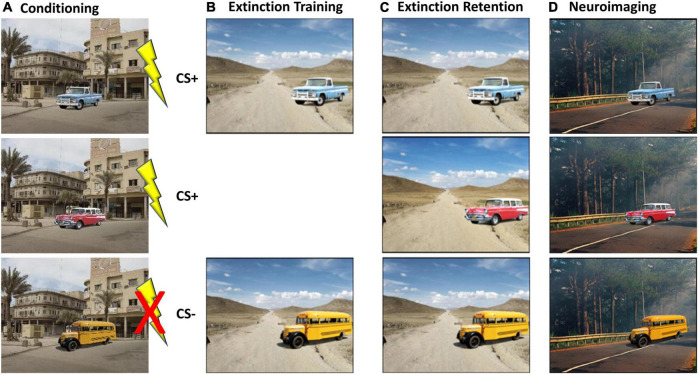
Overview of the modified fear-conditioning/fear-extinction protocol. **(A)** At baseline, participants were conditioned to fear two of three stimuli (i.e., blue truck and red car) by a mild but not painful electric shock (Pace-Schott et al., 2009), and skin conductance was measured. **(B)** On the same day, participants underwent extinction of the blue truck, but not the red car. Extinction was demonstrated by a reduction in skin conductance. **(C)** After 6 weeks of morning light treatment (blue or amber), participants returned to the lab and were shown the same stimuli again and skin conductance was measured. **(D)** Participants were shown the same stimuli, in a different visual context, while undergoing functional magnetic resonance imaging (fMRI).

During the baseline visit, participants underwent a modified version of a well-validated fear conditioning protocol (Milad et al., 2007; Pace-Schott et al., 2009; Marin et al., 2017). While the original version of the task utilized photographs of colored lamps on a desk (Milad et al., 2005), the current version was modified to be more appropriate to military PTSD settings. Specifically, the participant was first conditioned to fear two particular stimuli (e.g., a blue or red or yellow vehicle) in a specific context (e.g., city street in Baghdad Iraq), by providing a mild electric shock when the conditioned stimuli were shown, as shown in Figure 3A. A third stimulus (e.g., yellow bus) was never paired with the electric shock and served as a “non-reinforced CS” or CS–. Participants were randomized across 8 different stimulus/context conditions, counterbalancing vehicle color and conditions across CS conditions and presentation contexts, as well. Our participants showed a rapid acquisition of the conditioned fear response for the conditioned stimuli (e.g., red and blue vehicles), as evidenced by increased skin conductance and/or self-report indicating they expected a shock by the last two stimulus presentations during conditioning for the CS + S. Next, as shown in Figure 3B, the goal was to extinguish one conditioned stimulus (i.e., blue truck) by repeatedly showing the stimuli in a novel context (e.g., a dirt road in Afghanistan), but without any electric shock administered. After 16 trials where the stimuli were shown without any further shock, the skin conductance response (SCR) to the blue truck is expected to return to normal. Thus, at this phase of the task, the fear response to the blue truck has been successfully “extinguished” by the creation of a new “safety memory.” However, the red vehicle, which was previously paired with the electric shock, is never shown again in this new context, so it retains the saliency of the initial conditioned fear response. In this example, the yellow bus was never paired with a shock, so it is expected to continue to evoke very little SCR. After 6-weeks of light exposure therapy (BLUE or AMBER), participants returned to the lab and were shown the same stimuli again but without any new shock stimuli. Skin conductance was monitored throughout to assess the degree to which the safety memory was consolidated and retained subsequent to extinguishing (see Figure 3C).

#### Psychophysiological monitoring

##### Skin conductance

During the fear conditioning paradigm, skin conductance was continuously monitored at 37.5 Hz using disposable, MRI-safe 11-mm, Ag/AgCl sensors filled with isotonic paste attached 14 mm apart on the hypothenar surface of the left hand and recorded using the MP150 system with *Acqknowledge* acquisition software (BIOPAC Systems, Inc., Goleta, CA, United States). SCR was calculated for each trial as the mean skin conductance level in microSiemens (μS) during the last 2 s of context presentation, subtracted from the maximum skin conductance level during the 6 s of CS presentation. SCRs were square-root transformed; if the untransformed SCR was negative, the negative sign was retained after calculating the square root of the SCR’s absolute value (Orr et al., 2000). “Non-conditioners” were defined as those who exhibited less than 2 non-square-root transformed SCR responses to a CS + that equal to or exceeding 0.05 μS during the Fear Conditioning phase. Non-conditioners (8 Blue and 6 Amber) were excluded from analyses.

Outcome variables included SCR and Differential SCR (SCRd) equal to SCR to a CS + minus SCR to its ordinally corresponding CS-. Summary variables for the Fear Conditioning phase included a measure of differential “Conditionability” that was defined as the mean SCRd to all CS + s (excluding the first to each CS +). For the Extinction Learning phase, an Extinction Learning Index (EXTidx) was defined. EXTidx was calculated by subtracting mean SCR to the last four CS + E presentations from mean SCR to the first four CS + E presentations, dividing this value by the Maximum Conditioning CR, and multiplying by 100. The degree of extinction recall was represented by the Extinction Recall Index (ERI) calculated from the differential SCRs during the first two trials of the CS + E minus the first two trials of the CS + U during the extinction memory recall test. Difference scores greater than zero indicate greater responding to the CS + E, while difference scores equal to zero reflect no difference in responding between the CS + E and CS + U, and negative values reflect greater responding to the CS + U (Rabinak et al., 2013). The magnitude of extinction recall (ERM) was also quantified by subtracting the mean SCR of the first four CS + from the mean SCR for the first four CS + E during extinction recall and is representative of the same contrast used in the main neuroimaging analysis (CS + E > CS + ; Shvil et al., 2014).

##### Electrocardiogram

Participants also had ECG monitored for a 5-min resting period prior to fear conditioning, during which participants were instructed to sit quietly without talking or moving while focusing on a fixation cross positioned in front of them. ECG was acquired using a Zephyr Biopatch^1^ sampling at 1000 Hz. Off-line analysis was performed by extracting the interbeat interval (IBI) series from the raw digitized ECG signal using QRSTool Software (Allen et al., 2007). The extracted IBI series was then hand-corrected for artifacts such as ectopic, erroneous, and missed beats.

Data were processed using Matlab with parameters modeled on CMetX Cardiac Metric Software and using a “moving window” (Allen et al., 2007). The moving window comprised 16-s chunks that shift by 4 s at a time. The IBI series was converted to a time series sampled at 10 Hz with linear interpolation in order to estimate total heart-rate variability (Cook and Miller, 1992). The root mean square of successive differences (RMSSD), a time-domain measure proposed to quantify parasympathetic mediated autonomic control was also derived for use in subsequent analyses (Berntson et al., 2005; Kromenacker et al., 2018).

#### Neuroimaging

##### Fear conditioning extinction recall task

Figure 3D, shows that the final phase of the fear conditioning paradigm was to examine brain activation patterns to the same stimuli during functional magnetic resonance imaging (fMRI). An important consideration is that prior to the fMRI scan, the CS + U image (e.g., red vehicle) was also “extinguished” by presenting the CS + E and CS + U in the extinction context eight more times, as part of the standard fear conditioning protocol to ensure no latent adverse experiment effects related to a sustained conditioned fear response. Following the final extinguishing phase, participants reported not expecting a shock to any stimuli/context presentation administered. As shown in Figure 3D, while undergoing a later fMRI, participants were shown a new set of images that included the three previously seen target stimuli (i.e., blue truck, red car, yellow school bus), without any new shocks but in a completely novel situation. Prior studies have demonstrated adequate sleep is critical to context generalization following safety learning, in that the fear-conditioned response can return when the previous CS + stimuli are shown in a novel visual context (e.g., forest road). The stimuli were shown in a new visual context. Scanning occurred on a 3T Siemens Skyra MRI scanner. Contrasts were created that directly compared brain activation patterns from the previously extinguished stimuli (CS + E; blue truck) versus the never extinguished stimuli (CS + ; red car) and non-conditioned stimuli (CS–, yellow bus). These contrast maps were then compared at post-treatment between the BLUE and AMBER conditions.

##### Neuroimaging parameters

Neuroimaging data were collected using a 3T Siemens MAGNETOM Skyra using a 32-channel head coil. Head movement was restricted using foam cushions during image acquisition. We collected a high-resolution anatomical T1-weighted (T1w) MPRAGE (TR/TE/flip angle = 2100 msec., 2.33 ms, 12°) that consisted of 176 slices (256 × 256 matrix) with a slice thickness of 1 mm and voxel size of 1 mm × 1 mm × 1 mm. Functional images were acquired using a gradient echo T2*-weighted sequence (TR/TE/flip angle = 2000 ms, 25 ms, 90°). Before each scan, four images were acquired and discarded to allow longitudinal magnetization to reach equilibrium. The T1 and T2 images were collected in the same plane (whole brain acquisition; axial slices angled perpendicular to the AC-PC line). During the 9-min functional task, participants monitored a screen and were presented with the same CSs from the initial fear conditioning paradigm in a novel environmental context. Images were collected with the same slice thickness (3.125 mm, skip 1 mm; voxel size 3.125 × 3.125 × 3.125 mm) across 34 interleaved slices using head to foot phase encoding for a T2*-weighted BOLD EPI sequence (TR/TE/flip angle = 2.5 s/35 ms/90°).

### Statistical analysis

Hypotheses for repeated measures were tested, relative to main treatment effects, using 2 (BL, PL) × 2 (baseline, post-treatment) linear mixed models (estimated using REML and nloptwrap optimizer) for psychological outcome variables; to investigate interactions between treatment group, study phase, and potentially meaningful outcome measures. Sex and age were also included as covariates to account for theorized differences in symptomology. Dependent measures were log-transformed as necessary to improve normality and meet model assumptions (verified via AIC-criteria model comparison), and participants were included in the model as a random effect. Standardized parameters were obtained by fitting the model on a standardized version of the dataset, with 95% Confidence Intervals (CIs) and *p*-values computed using the Wald approximation. Results include Nakagawa’s Pseudo-*R*^2^ for marginal (only variances of the fixed components), as well as conditional (variance considered for both fixed and random effects). Analyses targeting interactions were performed using R (v4.1.0) with the lme4 package, and the report and sjtools packages were employed for model summarization. See Appendix 1 for full summaries of model coefficients reported below.

#### Fear conditioning statistical analysis

Skin conductance response data during Fear Conditioning, Extinction Learning, and Extinction Recall were analyzed using mixed Analysis of Variance (ANOVA). Included in all ANOVAs was the between-subjects factor for “Group” (Amber, Blue) and the within-subject factor CS Type (CS + and CS–) and Trial (number varied with phase and analysis, see section “Results”). For Fear Conditioning, a second within-subject variable, “Order” (CS + 1 and CS + 2), was added to the model. For Extinction Learning, Trial was replaced with “Trial Pair,” which averaged Extinction Learning trials in a pair-wise manner (Trial 1 and 2, 3 and 4… 15 and 16). During Extinction Recall, an additional within-subject variable, “CS + Type” (CS + E and CS + U) variable was added to the model. All pair-wise comparisons within the ANOVA model were made using means comparisons. Potential confounding effects of discrete (sex) or continuous variables PTSD severity (PCL-5 total, CAPS-5 severity), age, and maximum SCR to a CS + during Conditioning were tested by adding them to total models. Significance was set at *p* < 0.05 and the Greenhouse–Geisser correction was applied to all within-subject main effects and their interactions. Simple regression analyzed relationships between psychophysiological and subjective summary outcome measures, subjective and objective sleep variables, and psychometric measures. Two-sample *t*-tests compared BLT and ALT groups for each of the unitary indices (CondIdx, ExtIdx, ERI).

#### Image processing and statistical analysis

Image processing and statistical analysis were undertaken in SPM12 (Wellcome Department of Cognitive Neurology, London, United Kingdom^2^) following a standard pipeline that involved image realignment, unwarping, co-registration, normalization to Montreal Neurological Institute (MNI) coordinate space, spatial smoothing (6 mm full-width at half maximum), and reslicing to 2 × 2 × 2 mm voxels. A high pass filter (128 s cut-off period) was implemented to remove low frequency confounds, and the Artifact Detection Tool^3^ was used to remove motion artifacts and outlier scans that exceeded 3 SD in mean global intensity. At the individual level, a general linear model (GLM) was specified for contrasts that directly compared brain activation patterns from the previously extinguished stimuli (CS + E; e.g., blue truck) versus the never extinguished stimuli (CS + ; e.g., red car) and non-conditioned stimuli (CS-; e.g., yellow bus). These contrast maps were then compared at post-treatment between the BLUE and AMBER conditions. Based on prior research investigating functional brain activation during fear conditioning (Linnman et al., 2012), our analyses included regions of interest comprising a model of the fear neurocircuitry that included the following: the anterior cingulate cortex (ACC; a region associated with the expression of fear memories), the amygdala (a region associated with threat responding), and the insula (a region associated with the mediation of context threat). As the sample was underpowered relative to the targeted size identified in our power analysis, we employed a more liberal height threshold (*p* < 0.005, uncorrected), and utilized family wise error extent threshold correction (FWE cluster correction, *p* < 0.05, which resulted in *k* = 103 voxels, as indicated by SPM12). The first eigenvariate (a standard SPM output vector reflecting the signal intensity for each participant for that cluster) was extracted for secondary analyses.

## Results

### Structured clinical interview for DSM-V and clinician administered post-traumatic stress disorder scale for DSM-V

Participants showed a decline in PTSD symptoms and severity between baseline and post-treatment assessments. There was a strong effect of time on both PTSD severity, *beta* = *–0.62, 95% CI [–0.80, –0.44], t(154)* = *–6.67, p* < *0.001*, as assessed by the Clinician-Administered PTSD Scale (CAPS-5). There was also a decrease in PTSD symptoms, as assessed by the PTSD checklist (PCL-5), *beta* = *–0.38, 95% CI [–0.52, –0.24], t(155)* = *–5.24, p* < *0.001*. However, the effect of time was not qualified by a significant group x time interaction, *p*s > 0.290, suggesting that both groups improved in PTSD symptoms and severity.

### Sleep

Results showed that subjective sleep tended to improve between baseline and post-treatment. However, this improvement was not qualified by significant interaction effects and both groups tended to show similar improvements. Individuals in both the ALT and BLT reported improved sleep as measured by lower PSQI scores (indicating fewer symptoms of disrupted sleep), *beta* = *–0.23, 95% CI [–0.37, –0.09], [t(151)* = *–3.21, p* = *0.001*] and higher FOSQ scores *beta* = *0.07, 95% CI [0.00, 0.14], t(154)* = *2.16, p* = *0.031*. Both groups also reported lower levels of insomnia severity as measured by the ISI *{beta* = *–0.38, 95% CI [–0.54, –0.21], t(157)* = *–4.47, p* < *0.001*} and nightmares as measured by the DDNSI *{beta* = *–0.16, 95% CI [–0.30, 0.03], t(142)* = *–2.32, p* = *0.020*}. Changes in daytime sleepiness as measured by the ESS were not significant *{beta* = *–0.13, 95% CI [–0.29, 0.04], t(155)* = *–1.52, p* = *0.128*} and none of the group x time interactions were significant, all *p*s > 0.130. Outcomes based on actigraphy data are discussed elsewhere, see Killgore et al. (2022) in review.

### Fear conditioning and extinction learning

**FIGURE 4:**
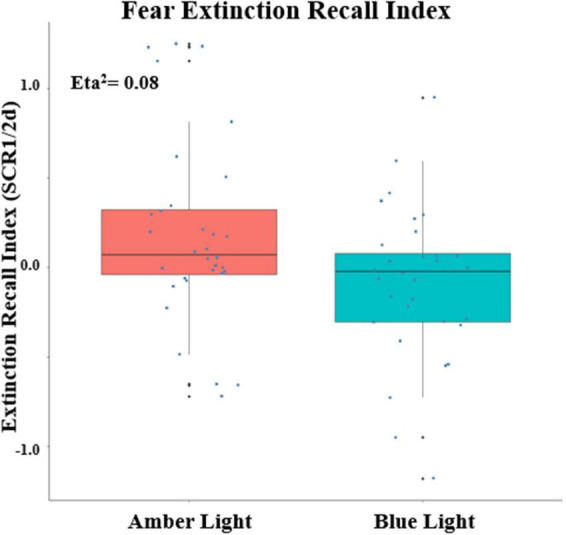
Fear Extinction Index (ERI) calculated from the differential SCRs during the first two trials of the CS + E minus the first two trials of the CS + U during the extinction memory recall test, Positive values indicate greater recall as measured by Skin Conductance Response (SCR) at post-treatment. The upper and lower “hinges” correspond to the first and third quartiles (the 25th and 75th percentiles) and lines correspond the median value. SCR1/2d: differential SCR.

Because there was a main effect on SCR of being a conditioner vs. non-conditioner [*F(1,74)* = *6.11, p* = *0.0158*, η*_*p*_^2^* = 0.0762], the 8 BLT- and 6 ALT-Group non-conditioners were excluded from further analyses. This left a total sample size of 68 with 34 each in the ALT and BLT groups and 23 males and 45 females. Lack of baseline differences in SCR between Groups during Fear Conditioning was confirmed by there being no main effect of Group or interaction of Group with Order, CS Type or Trial or higher order interactions of Group with these factors (all *p*’s > 0.22). Similarly, at Extinction Learning there was no main effect of Group or interactions of Group with CS Type, Trial or higher-order interactions (all *p*’s > 0.26). SCR measurements confirmed that differential conditioning was acquired and extinguished. For Fear Conditioning there was a significant Order × CS Type X Trial interaction *[F(6,342)* = *2.76, p* = *0.0172*, η*_*p*_^2^* = *0.0462]*. Similarly, at Early Extinction there was a significant CS Type x Trial interaction *[F(7,462)* = *2.98, p* = *0.0082*, η*_*p*_^2^* = *0.0432]*. Among unitary indices, ALT and BLT groups did not differ in maximum SCR to a CS + (at Conditioning) or in CondIDx and ExtIdx at baseline (*p*’s > 0.42). However, significant group effects were observed in ERI at the end of treatment *{F(1, 60)* = *5.08, p* = *0.028;* η*_*p*_^2^* = *0.08, 90% CI [4.69e-03, 0.20]}* (see Figure 4); as well as ERM *{F(1, 60)* = *4.33, p* = *0.042;* η*_*p*_^2^* = *0.07, 90% CI [1.40e-03, 0.19]}*. In addition, none of these indices correlated with PCL-5 total or CAPS-5 severity scores (all *p*’s > 0.23) with the exception of a positive correlation between CAPS-5 severity and maximum SCR to a CS + at Conditioning (*R* = 0.252, *p* = 0.045).

**FIGURE 5:**
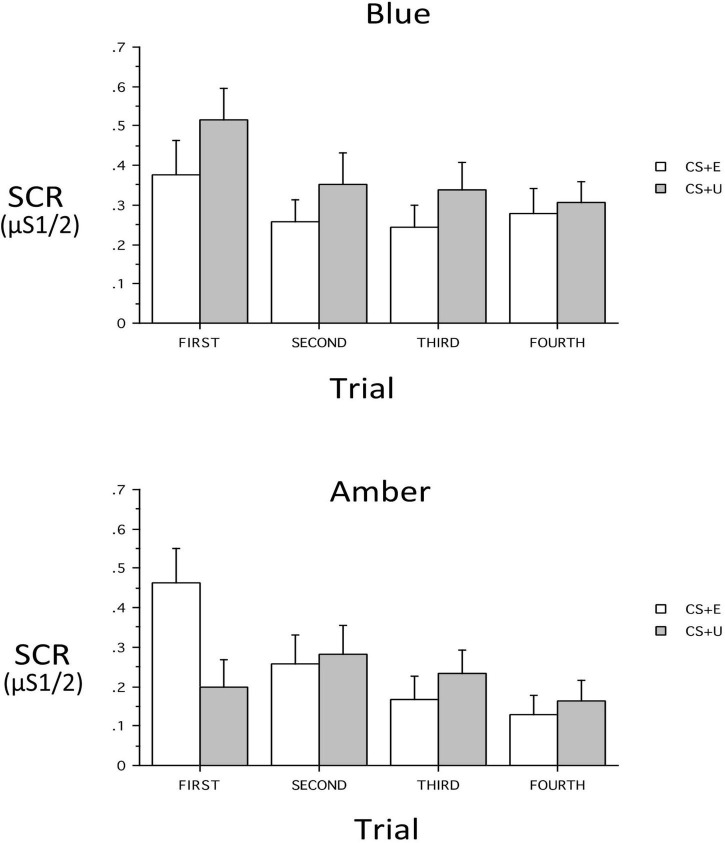
Skin conductance responses (SCR) across trials for the blue light treatment-BLT **(top)** and amber light placebo treatment-ALT **(bottom)** for the extinguished (CS + E) versus unextinguished (CS + U). The BLT condition led to reduced SCR to extinguished relative to unextinguished stimuli (especially on the first trial), while the opposite was true for the amber placebo condition. μS, microSiemens. Error bars represent standard error of the mean.

At Extinction Recall, a main effect of CS_Type (CS + vs. CS–) indicated retention of differential conditioning. However, lack of a main effect of CS + Type (CS + E vs. CS + U) indicated that the distinction between the extinguished and unextinguished CS + was not retained across all subjects. At Extinction Recall, there was no main effect of Group, nor were there interactions of Group with CS_Type, CS + Type, or Trial (all *p*’s > 0.13) or 3- or 4-way interactions with these within-subject factors with the exception of a Group × CS + Type × Trial interaction *[F(3,186)* = *3.48, p* = *0.026*, η*_*p*_^2^* = *0.0531]*. Therefore the 2 Groups were examined individually. In the BLT Group, there were no significant main effects or interactions. However, CS + -Type, CS_Type and Trial main effects appeared as trends (*p* = 0.089, 0.089, and 0.068 respectively) with absolute value of SCR for CS + > CS- and CS + U > CS + E. In the ALT Group, there was no main effect of CS + Type, but significant main effects for CS_Type *[F(1,31)* = *11.86, p* = *0.0017*, η*_*p*_^2^* = *0.277]* and Trial *[F(3,93)* = *6.30, p* = *0.0025*, η*_*p*_^2^* = *0.169]*. There was, in addition, a CS + x Trial interaction *[F(3,93)* = *4.05, p* = *0.0184*, η*_*p*_^2^* = *0.116]*. This interaction resulted from a significantly greater SCR in the first trial to the CS + E than to the CS + U [*p* = 0.0025), but not in Trials 2–4 (all *p*’s > 0.38). In contrast, in the BLT group, despite lack of an overall interaction (*p* = 0.666), there was a greater SCR in the first trial to the CS + U than to the CS + E (*p* = 0.0464) while in all other trials the CS + Type did not significantly differ (all *p*’s > 0.15). In each trial the absolute value of CS + U was greater than CS + E, resulting in the above-noted a trend for main effect of CS + Type (CS + U > CS + E) (see Figure 5).

**FIGURE 6:**
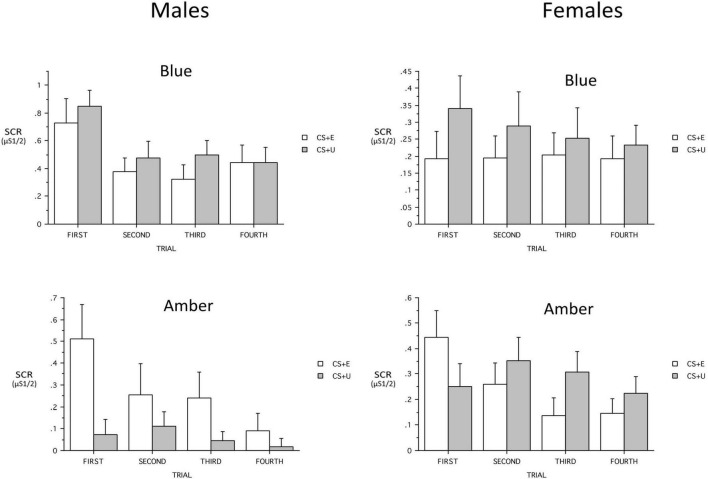
Sex × light color interactions on skin conductance responses (SCR) across trials for the blue light treatment-BLT (top) and amber light placebo treatment-ALT (bottom) for the extinguished (CS + E) versus unextinguished (CS + U). μS, microSiemens. Error bars represent standard error of the mean. Error bars represent standard error of the mean and Y-axis scales are heterogeneous.

When added ANOVA models, the continuous variables that included PTSD severity (PCL-5 total, CAPS-5 severity), age, and maximum SCR to a CS + during Conditioning, showed no main effects and only a few interactions with Group. Age showed a three-way interaction with Group and CS_Type *[F*(1,60) = 5.73, *p* = 0.0198, η*_*p*_^2^* = 0.087*]* and the Group × CS_Type trended [*F*(1,60) = 3.84, *p* = 0.055, η_*p*_^2^ = 0.06]. Similarly, when maximum SCR to a CS + during Conditioning was added, it showed a three-way interaction with Group and CS_Type *[F(1,57)* = *8.80, p* = *0.004*, η*_*p*_^2^* = *0.087]* and the Group × CS_Type interaction was significant *[F(1,57)* = *4.34, p* = *0.0418*, η*_*p*_^2^* = *0.134]*. In both cases, the difference between the (combined) CS + s and the CS- was greater in ALT. However, when Sex was added to the model, there was a significant Group x Sex interaction *[F(1,60)* = *5.10, p* = *0.027*, η*_*p*_^2^* = *0.079]* as well as Group × CS + Type *[F(1,60)* = *4.32, p* = *0.0420*, η*_*p*_^2^* = *0.067]* and Group × CS_Type × Sex interaction *[F(1,60)* = *4.03, p* = *0.0493*, η*_*p*_^2^* = *0.063].* The Group × CS + Type × Trial interaction was a trend *[F(2,180)* = *2.85, p* = *0.053*, η*_*p*_^2^* = *0.045]* (see Figure 6). When the 23 males were examined separately, there was a significant Group main effect *[F(1,18)* = *8.12, p* = *0.0107*, η*_*p*_^2^* = *0.311*, BLT larger] and the Group × CS + Type Interaction demonstrated a trend *[F(1,18)* = *4.31, p* = *0.0525*, η*_*p*_^2^* = *0.193]*. The Group × CS + Type × Trial interaction observed in the total sample was absent (*p* = 0.30). Among males there was no CS + Type × Trial interaction in either the 10 ALT or the 13 BLT males (*p* = 0.26 and 0.51 respectively). The 45 females were examined separately among 24 ALT and 21 BLT, and there was a Group × CS_Type interaction *[F(1,42)* = *4.15, p* = *0.048*, η*_*p*_^2^* = *0.09]* and the Group × CS + Type × Trial was a trend *[F(3,126)* = *2.68, p* = *0.07*, η*_*p*_^2^* = *0.06].* Among ALT females there was a CS + Type × Trial interaction trend *[F(3,66)* = *2.83, p* = *0.06*, η*_*p*_^2^* = *0.114; CS* + *E larger]* whereas no associated interaction was found in BLT females (*p* = 0.66).

### Root mean square of successive differences results

In total, 14 subjects had unusable ECG recordings due to noise or data corruption, while another 11 participants had to be excluded due to the presence of ectopic heartbeats. This yielded 57 cases with complete pre- and post-treatment ECG recordings. No significant time or group x time interactions were observed for RMSSD, *beta* = *0.03, 95% CI [–0.29, 0.34], t(112)* = *0.17, p* = 0.863.

### Neuroimaging

**FIGURE 7:**
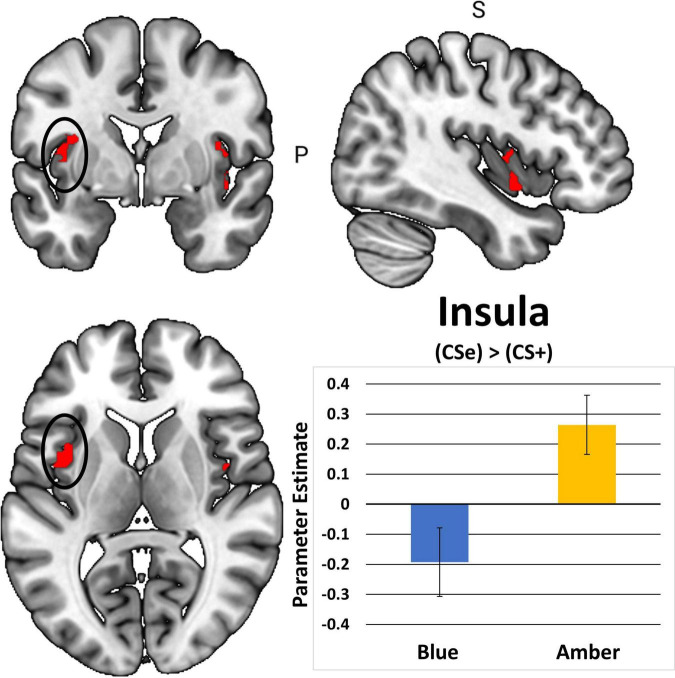
The contrast between extinguished (CS + E) versus unextinguished (CS +) was compared between the BLUE and AMBER conditions at post-treatment. Participants in the amber light condition showed a significant increase of activation in the bilateral insula areas, with the left insula activation (circle) surviving FWE correction. Error bars represent standard error of the mean.

In total, 1 participant did not demonstrate fear-based learning, 9 participants had unusable scans, and an additional 18 participants were excluded due to excessive movements during the post-treatment scan. This yielded 48 cases with complete post-treatment scans. Table 2 shows that BLT was associated with significant differences in responses to previously extinguished stimuli relative to ALT. Moreover, as seen in Figure 7, BLT resulted in a significant decrease in activation responses within the left insular cortex relative to ALT, which was significant after cluster correction for multiple comparisons. Overall, this suggests that when viewing previously feared and then extinguished stimuli, ALT participants responded with increased activation of somato-visceral brain regions involved in anxiety, while BLT participants showed a reduction of responses in this area.

**TABLE 2:** Fear conditioning recall activation clusters.

Region of Interest	Cluster	Peak	MNI	Peak Region
	
	Size (voxels)	equiv Z	*x y z*	
Insula	49	3.53	38 4 14	Right Insula
	108	3.43	–38 6 8	Left Insula*
	32	3.4	44 4 –10	Right Insula
	4	2.89	32 28 –4	Right Insula
Cingulate Cortex				
	41	3.74	16 –24 42	Right MCC
	15	3.39	10 46 26	Right ACC
	8	2.8	0 40 20	ACC
Amygdala				
	4	2.82	22 0 –18	Right Amygdala

All voxels significant at p < 0.005 (uncorrected); *Indicates cluster survived cluster-based Family Wise Error Correction FWEc. MNI, Montreal Neurologic Institute coordinates.

### Full sample zero-order correlations

**FIGURE 8:**
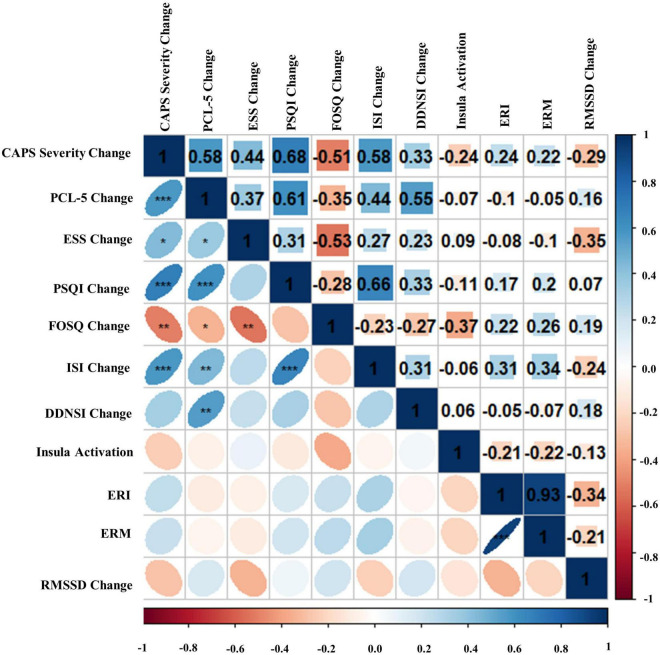
Bivariate Pearson correlations were performed across Blue Light Treatment (BLT) subjects with correlation coefficients in the upper portion of the matrix and significant correlations identified in the lower portion of the matrix. Change represents residualized change scores. *p < 0.05, **p < 0.01, ***p < 0.001. Clinician-Administered PTSD Scale for DSM-5 (CAPS-5), PTSD Checklist for DSM-5 (PCL-5), Epworth Sleepiness Scale (ESS), Pittsburgh Sleep Quality Index (PSQI), Functional Outcomes of Sleep Questionnaire (FOSQ), Insomnia Severity Index (ISI), Disturbing Dreams and Nightmares Severity Index (DDNSI), Extinction Recall Index (ERI), Extinction Recall Magnitude (ERM), Root Mean Square of Successive Differences (RMSSD).

**FIGURE 9:**
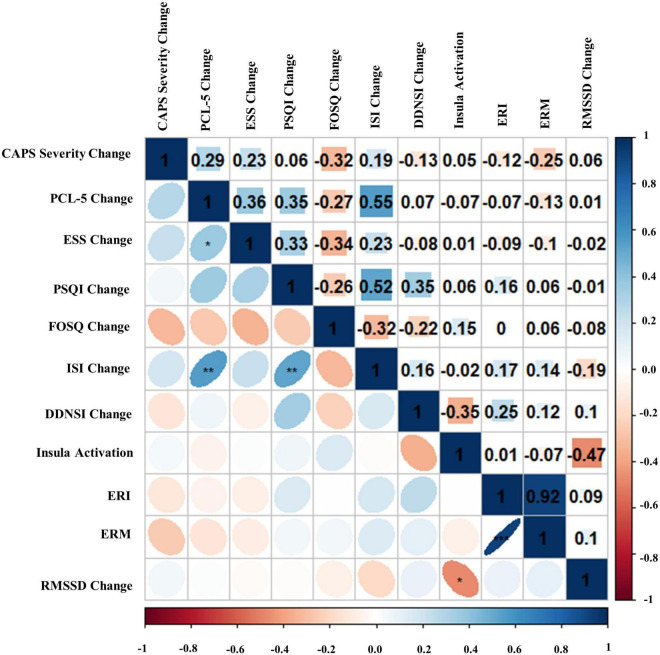
Bivariate Pearson correlations were performed across Amber Light Treatment (ALT) subjects with correlation coefficients in the upper portion of the matrix and significant correlations identified in the lower portion of the matrix. Change represents residualized change scores. *p < 0.05, **p < 0.01, ***p < 0.001. Clinician-Administered PTSD Scale for DSM-5 (CAPS-5), PTSD Checklist for DSM-5 (PCL-5), Epworth Sleepiness Scale (ESS), Pittsburgh Sleep Quality Index (PSQI), Functional Outcomes of Sleep Questionnaire (FOSQ), Insomnia Severity Index (ISI), Disturbing Dreams and Nightmares Severity Index (DDNSI), Extinction Recall Index (ERI), Extinction Recall Magnitude (ERM), Root Mean Square of Successive Differences (RMSSD).

Bivariate correlations assessing relationships among BLT (see Figure 8) and ALT (see Figure 9) for residual change scores of PTSD severity, sleep outcomes, RMSSD, as well as fear responding across either treatment group. For BLT, decreased PTSD symptoms and symptom severity were associated with improvements in sleep quality, daytime sleepiness, functional outcomes, nightmares, as well as insomnia; while associations for ALT were only observed relative to decreased symptoms and two metrics of daytime sleepiness and insomnia. Relative to autonomic and neural outcomes, individuals in the ALT demonstrated an association between decreased RMSSD across the treatment period and activation in the insula. No associations between autonomic and neural outcomes with behavioral metrics for BLT were observed (see Figure 8). However, observed associations did not remain significant after Bonferroni correction for multiple comparisons for either group.

## Discussion

In this study, we investigated the hypothesis that exposure to blue-wavelength light for 30-min each morning for 6-weeks would enhance sleep and the retention of fear extinction memory during a classical fear conditioning paradigm. Based on prior findings, we hypothesized that BLT would be associated with improvements in sleep, PTSD symptomology, and related neurobiological outcomes. Overall, we found support for this hypothesis, although with some qualifications. We discuss these findings and their implications in detail below.

### Primary hypotheses

First, we hypothesized that daily morning blue-wavelength light exposure would lead to associated improvements in self-reported sleep and reduced PTSD symptom severity relative to amber placebo treatment. This hypothesis was supported, as individuals receiving BLT demonstrated significant improvements in the severity of their PTSD symptoms, which were associated with improvements across the indices measuring components of sleep and dysfunction. Contradictory to expectations, individuals in the ALT group also demonstrated significant decreases in PTSD symptom severity; however, these changes were not associated with improvements in subjective sleep outcomes. This may be because the behavioral component of the intervention, requiring participants to wake up before 11 a.m. every day and engage in a specific task (light exposure for 30-min), may have served to stabilize their circadian rhythm relative to pretreatment for both groups. However, changes in PTSD severity for individuals receiving ALT were independent of functional outcomes related to sleep, suggesting that BLT directly influences the reciprocal nature of sleep dysfunction and PTSD (see Figure 8), as opposed to the two domains independently.

Second, we hypothesized that individuals receiving daily morning blue-wavelength light exposure would demonstrate enhanced retention of extinction learning in patients with PTSD, as evidenced by changes in autonomic and neural responses. We found that relative to BLT, individuals in the ALT demonstrated increased responding, as measured by SCR, for stimuli they had demonstrated a previous safety learning response after initial fear conditioning. The chief difference between the BLT and ALT groups at Extinction Recall was that, in the BLT group, absolute values of the SCR to conditioned and un-extinguished stimuli (CS + U) were consistently higher than the conditioned and extinguished stimuli (CS + E) as would be expected from retained extinction memory (see Figure 5). In contrast, in the ALT group, the opposite was the case (see Figure 5). Whereas this interaction (Group × CS + Type) did not reach trend or significance levels across all 4 trials, in the first trial, SCR to the CS + E significantly exceeded that to the CS + U in the ALT group whereas SCR to the CS + U significantly exceeded that to the CS + E in the BLT group. If one assumes new (re)extinction immediately begins to take place during Extinction Recall, then this first trial takes on special significance as purely reflecting extinction recalled rather a combination of prior extinction memory and new extinction being learned. As there was a significant Group × Sex interaction and the Group × CS + Type × Trial interaction demonstrated a trend, the two groups within each of the two sexes were examined individually. Although the CS + Type × Trial interaction did not reach significance in either sex alone, a similar Group difference (i.e., SCR to CS + U > CS + E in BLT, CS + E > CS + U in ALT) appeared in each sex individually especially in the first trial (see Figure 6). Although one might expect the CS + Type × Trial interaction to be present in ALT Males (see Figure 6), the low sample size of this subgroup (*N* = 10) likely prevented this. Thus, the ALT group reacted to the previously extinguished stimulus not only as if was unextinguished, but to a greater degree than the unextinguished stimulus. The BLT group, however, reacted to previously extinguished stimuli as expected (i.e., diminished fear response). This suggests that, although differential conditioning was retained across all subjects (CS + > CS–), retention of stimulus-specific extinction memory occurred only in the BLT group. This was further evidenced by increased activation in the insular region of the brain for individuals that received ALT when they were shown the previously feared stimuli in a novel context following a second safety learning period. We also found that for individuals in the ALT group, this increased insular responding was associated with diminished autonomic regulation at rest, as measured by RMSSD, while no such association was observed for individuals in the BLT group.

### Considerations

No known studies have examined the neurobiological or autonomic correlates of symptom improvement in patients with PTSD following blue light exposure therapy. Prior work has demonstrated BLT as an effective treatment for sleep disruption following mild traumatic brain injury (Raikes et al., 2020; Killgore et al., 2022), however, the use of this type of treatment as a means of improving PTSD symptoms is novel. Recent pilot studies have examined the potential to use light-based treatments to facilitate recovery from PTSD and have shown promising results in preliminary trials. For instance, Zalta et al. (2019) used a commercial head-mounted device that presented green wavelength light and found that the bright light exposure condition was better than the dim light control condition at improving symptoms of PTSD and depression. Similarly, Youngstedt et al. (2021) used a bright white light device compared to an inert control condition (i.e., an ion generator) and found that 4-weeks of daily light exposure for 30-min was effective at improving symptoms of PTSD and general clinical impression ratings. Those samples were small to modest in size and included significantly fewer controls than in the present study. Here, we find that under more tightly controlled conditions, with a similar bright light control condition, we were able to find significant improvements in clinical outcomes, fear conditioning, and neuroimaging activation patterns. Sleep-focused interventions have also been proposed as a means of augmenting current TBI and PTSD treatment protocols (Gilbert et al., 2015). As the two disorders often co-occur and also present with co-morbidities, such as depression (Tanev et al., 2014), BLT may serve to meet future needs as an effective treatment that positively influences factors predictive of treatment success. The present study provides clear evidence that both functional and neurobiological changes are associated with changes in sleep, fear extinction memory consolidation, and PTSD symptoms from pre- to post-treatment for individuals receiving BLT.

A growing body of work demonstrates daily BLT can be used as a potent non-pharmacological intervention to improve sleep and mood symptoms. Depressive symptoms associated with seasonal affective disorder have often been a target for BLT interventions, with multiple studies demonstrating its efficacy for inducing clinically significant changes in symptom presentation (Glickman et al., 2006; Strong et al., 2009; Chang et al., 2018). Furthermore, PTSD and depression, while distinct, have numerous overlapping distress components (Post et al., 2011). Here we demonstrate that the well-established use of BLT for decreasing mood symptoms relating to depression can be extended to individuals suffering from PTSD. This may be, in part, due to the underlying neurobiological relationship between sleep and emotional wellbeing (Goldstein and Walker, 2014). Light therapy as a treatment for mood disorders is becoming increasingly accepted, and the current study is consistent with calls for standard approaches and rigorous study designs relative to its utility (Golden et al., 2005). However, more work is necessary to identify individual features that contribute to mood-related outcomes in BLT, as well as, how best to optimize a standardized intervention approach for delivering light therapy.

A key finding is that participants receiving ALT showed a return of learned fear responses, while BLT led to a retention of learned extinction. A resurgence of fear-based responding is a hallmark symptom in PTSD and findings suggest that increased sleep quality and decreased PTSD symptom severity via BLT during the intervening weeks led to greater consolidation of the fear extinction memory. Prior work demonstrates acute exposure to blue light augments memory consolidation and working memory (Alkozei et al., 2016b,2017b), however, those studies also demonstrated differences in memory effects relative to sex, and the present study revealed significant sex differences in extinction recall. Females in the ALT group demonstrated increased responding to the previously extinguished stimuli at the first trial relative to the unextinguished stimuli, while subsequent trials demonstrated the expected relationship in SCR (CS + U > CS + E). However, males in the ALT group demonstrated greater SCR to the CS + E stimuli compared to the CS + U stimuli across all four initial trials during fear recall. The current observations are consistent with prior work that has demonstrated sex differences in fear conditioning and recall (Baran et al., 2009; Velasco et al., 2019). Of interest, females with PTSD demonstrate greater acquisition of conditioned fear relative to men, and findings regarding sex differences in this patient population are not entirely clear (Inslicht et al., 2013), with other work demonstrating associations between pre-acquisition stress and increased fear learning in men and mixed findings in females (Peyrot et al., 2020). While the sex-based differences in fear recall observed in the current study are consistent with previous findings, future work is necessary to identify how sex influences BLT treatment outcomes for individuals with PTSD.

The finding of decreased insular responses to previously extinguished stimuli in the BLT group is particularly enlightening. Prior work has found that insula reactivity is related to fear conditioning and greater SCR (Critchley et al., 2000; Linnman et al., 2012; Fullana et al., 2016; Seo et al., 2022), which is consistent with findings we report here. Insula reactivity has been demonstrated to increase relative to uncertainty-related expectations, which may have been facilitated by presenting the fear-conditioned stimuli in a novel context during the neuroimaging task (Sarinopoulos et al., 2010). Furthermore, REM sleep dysfunction is associated with a lack of generalization relative to safety learning, and eliciting this deficit in safety learning was the main consideration relative to the novel task-based neuroimaging design (Pace-Schott et al., 2015b). It is also important to note that extinction learning was facilitated for all stimuli prior to neuroimaging, as both the CS + E and CS + U are extinguished during extinction recall as part of the established fear conditioning protocol after which our procedures were modeled, which may be why observable differences were not observed in the ACC or amygdala areas of the cortex. Prior studies demonstrate that autonomic dysfunction is associated with PTSD and that emotional reactivity and regulation become decoupled relative to HRV and insular activity (Thome et al., 2017; Schneider and Schwerdtfeger, 2020). This may be a driving factor in the association between diminished RMSSD across the intervention period for ALT relative to increased insular reactivity to previously feared stimuli.

Overall, these findings suggest that BLT was effective at sustaining fear extinction memory relative to ALT. This is critical to recovery from PTSD, as several prominent exposure treatments for this disorder are based extensively on principles of fear extinction (Paredes and Morilak, 2019). As fear conditioning is often cited as a key pathogenic mechanism in PTSD (Rauch et al., 2006), anything that augments fear extinction consolidation, such as BLT, would logically decrease the likelihood of PTSD subsequent to a traumatic event/intervention (Linnman et al., 2012). The current results suggest that BLT may potentially facilitate treatment gains from exposure-based therapies through non-circadian effects of blue light and melanopsin, as well as by stabilizing the circadian clock and improving sleep in a manner that promotes consolidation of extinction-based memories (Lazzerini Ospri et al., 2017). These initial findings are encouraging and suggest the need for further research to delineate the factors that contribute to positive outcomes. Future work would benefit by identifying the genetic, psychophysiological, and other individual difference factors that could allow greater precision in treatment application. Studies utilizing polysomnographic monitoring to better assess specific changes across multiple sleep parameters will also be particularly important in future investigations. Additional work will also be necessary to determine the specific timing, duration, and wavelengths that are most effective in producing changes in circadian timing and clinical outcomes.

### Limitations

Several limitations should be considered when interpreting the results of this study. The study sample was relatively small and multiple confounds (attrition, lack of fear learning, data corruption) led to many of the presented analyses being underpowered. Further work will be necessary to replicate and build on the current findings and observations relative to the effects of 6 weeks of morning BLT. It should be noted that these correlations do not indicate causal relationships between sleep and symptom improvement. As sleep and clinical symptomology are tightly intertwined, it is possible that improvements to PTSD, anxiety, and depression symptoms may have led to improvements to sleep, or that there were bidirectional effects. However, regardless of the precise directional relationship involved, the present results highlight the importance of sleep in the continuation of psychopathology and/or symptomatic improvements.

Upon the conclusion of the study, participants were asked whether they thought they had been assigned to the active or placebo treatment, and to indicate their confidence in their guess. The majority of participants believed that they had been assigned to the active condition, but this was also equally distributed between actual treatment assignments. The participants assigned to the BLT and ALT were also similarly confident in their guess, *t*(78) = 0.56, *p* = 0.576, *d* = 0.13, with both BLT (*M* = 3.63, *SD* = 1.05) and ALT (*M* = 3.48, *SD* = 1.38) groups being between 50 and 75% confident in their assessments. This suggests that participants did not have insight as to whether they were receiving the active or placebo treatment.

It is important to highlight that our placebo treatment, ALT, showed evidence of active effects. Amber light has previously been used as a placebo therapy (Raikes et al., 2020; Killgore et al., 2022), but further work will need to be done on whether it also significantly impacts sleep. Future research should consider adding an inactive no light condition to fully separate the effects of different light exposures on sleep and improvements to mental health. Another possible explanation for the consistent improvements is that the interventions may have been associated with better sleep and mental health for reasons other than the effects of active light components on sleep or brain function. For example, the act of using the lightbox, regardless of wavelength, may have aided in the development of a daily routine, which could provide patients with PTSD with a sense of control and purpose, as well as establish a more consistent daily rhythm through other zeitgebers (e.g., eating; social contact). Similarly, filling out sleep diaries every day may have helped participants become more aware of their sleep, mood, and day-to-day factors that influence sleep. Future research will be necessary to further investigate these possibilities, and to identify the specific parameters of light exposure (e.g., length of the treatment phase, light exposure duration) that will optimize treatment gains.

## Conclusion

The present study examined the association between 6 weeks of daily morning blue-wavelength light relative to amber-wavelength light placebo treatment. Overall, we found support for the hypothesis that improvements in sleep would be linearly correlated with improvements in PTSD symptom severity. Furthermore, the findings from the fear extinction task support the global hypothesis that BLT improves autonomic reactivity and brain function in a manner that aids in fear extinction/safety memory, even across longer durations of time than this type of paradigm is typically employed. This is especially important, as safety learning is critical to recovery and several prominent evidence-based treatments for PTSD are based extensively on principles of fear extinction. These results suggest that BLT is a promising non-pharmacological intervention that may potentially facilitate the retention of treatment gains from exposure-based therapies by resetting the circadian clock and improving sleep in a manner that promotes consolidation of extinction memory. Future work will need to identify the clinical significance of these outcomes as well as their temporal effect, but these results suggest that BLT may be useful for improving sleep and consolidating safety learning outcomes in a manner that promotes the reduction of sleep-related complaints and PTSD symptoms.

## Data availability statement

The raw data supporting the conclusions of this article will be made available by the authors, without undue reservation.

## Ethics statement

The studies involving human participants were reviewed and approved by The Institutional Review Board of the University of Arizona College of Medicine, The U.S. Army’s Human Research Protections Office. The patients/participants provided their written informed consent to participate in this study.

## Author contributions

WK: study conception and design. WK, JV, AB, ND, and SE: data collection. WK, JV, and EP-S: analysis and interpretation of results. JV, WK, EP-S, AB, ND, and SE: draft manuscript preparation. All authors contributed to the article and approved the submitted version.

## Footnotes

1
https://www.zephyranywhere.com/media/download/zephyr-performance-biopatchhp-brochure.pdf

2
http://www.fil.ion.ucl.ac.uk/spm

3
http://www.nitrc.org/projects/artifact_detect/
