# Supplementary material for: Morning blue light treatment improves sleep complaints, symptom severity, and retention of fear extinction memory in post-traumatic stress disorder
Source: Front Behav Neurosci. 2022 Sep 12;16:886816. doi: 10.3389/fnbeh.2022.886816 (PMC9510714; doi:10.3389/fnbeh.2022.886816)
Supplement: Supplementary file 1 [file Data_Sheet_1.docx]

Appendix 1) Statistical Model Coefficients

4.1 PTSD Results

4.1.1

| **Clinician-Administered PTSD Scale for DSM-5** **(CAPS) Severity** | | | |
| --- | --- | --- | --- |
| *Predictors* | *Estimates* | *CI* | *p* |
| Intercept | 3.32 | 2.95 – 3.68 | **<0.001** |
| Sex | -0.17 | -0.37 – 0.03 | 0.094 |
| Age | 0.01 | -0.00 – 0.02 | 0.279 |
| Study Phase | -0.62 | -0.80 – -0.44 | **<0.001** |
| Group | 0.09 | -0.14 – 0.31 | 0.453 |
| Study Phase * Groupt | 0.13 | -0.11 – 0.38 | 0.29 |
|  |  |  |  |
| **Random Effects** |  |  |  |
| σ^2^ | 0.16 |  |  |
| τ_00_ _participantid_ | 0.1 |  |  |
| ICC | 0.39 |  |  |
| N _participantid_ | 82 |  |  |
| Observations | 162 |  |  |
| Marginal R^2^ / Conditional R^2^ | 0.252 / 0.546 |  |  |

4.1.2

| **PTSD Checklist for DSM-5** **(PCL-5) Symptoms** | | | |
| --- | --- | --- | --- |
| *Predictors* | *Estimates* | *CI* | *p* |
| Intercept | 3.52 | 3.19 – 3.84 | **<0.001** |
| Sex | -0.13 | -0.31 – 0.06 | 0.175 |
| Age | 0.01 | -0.00 – 0.02 | 0.055 |
| Study Phase | -0.38 | -0.52 – -0.24 | **<0.001** |
| Group | 0.03 | -0.17 – 0.22 | 0.769 |
| Study Phase * Groupt | -0.06 | -0.25 – 0.14 | 0.565 |
|  |  |  |  |
| **Random Effects** |  |  |  |
| σ^2^ | 0.1 |  |  |
| τ_00_ _participantid_ | 0.1 |  |  |
| ICC | 0.51 |  |  |
| N _participantid_ | 82 |  |  |
| Observations | 163 |  |  |
| Marginal R^2^ / Conditional R^2^ | 0.205 / 0.609 |  |  |

4.2. Sleep Results

4.2.1

| **Pittsburgh Sleep Quality Index (PSQI) Total Score** | | | |
| --- | --- | --- | --- |
| *Predictors* | *Estimates* | *CI* | *p* |
| Intercept | 1.9 | 1.60 – 2.20 | **<0.001** |
| Sex | 0.02 | -0.14 – 0.19 | 0.765 |
| Age | 0.01 | -0.00 – 0.02 | 0.09 |
| Study Phase | -0.23 | -0.37 – -0.09 | **0.001** |
| Group | 0.1 | -0.08 – 0.28 | 0.275 |
| Study Phase * Groupt | 0.01 | -0.18 – 0.20 | 0.918 |
|  |  |  |  |
| **Random Effects** |  |  |  |
| σ^2^ | 0.09 |  |  |
| τ_00_ _participantid_ | 0.07 |  |  |
| ICC | 0.45 |  |  |
| N _participantid_ | 81 |  |  |
| Observations | 157 |  |  |
| Marginal R^2^ / Conditional R^2^ | 0.113 / 0.512 |  |  |

4.2.2

| **Functional Outcomes of Sleep Questionnaire (FOSQ)**  **Total Score** | | | |
| --- | --- | --- | --- |
| *Predictors* | *Estimates* | *CI* | *p* |
| Intercept | 2.65 | 2.46 – 2.84 | **<0.001** |
| Sex | 0.19 | 0.08 – 0.30 | 0.001 |
| Age | 0 | -0.01 – 0.00 | 0.549 |
| Study Phase | 0.07 | 0.01 – 0.14 | **0.031** |
| Group | 0.03 | -0.07 – 0.14 | 0.532 |
| Study Phase * Groupt | -0.06 | -0.15 – 0.03 | 0.189 |
|  |  |  |  |
| **Random Effects** |  |  |  |
| σ^2^ | 0.02 |  |  |
| τ_00_ _participantid_ | 0.04 |  |  |
| ICC | 0.67 |  |  |
| N _participantid_ | 82 |  |  |
| Observations | 162 |  |  |
| Marginal R^2^ / Conditional R^2^ | 0.117 / 0.710 |  |  |

4.2.3

| **Insomnia Severity Index (ISI) Total Score** | | | |
| --- | --- | --- | --- |
| *Predictors* | *Estimates* | *CI* | *p* |
| Intercept | 2.27 | 1.82 – 2.71 | **<0.001** |
| Sex | 0 | -0.25 – 0.25 | 0.999 |
| Age | 0.01 | -0.00 – 0.02 | 0.158 |
| Study Phase | -0.38 | -0.54 – -0.21 | **<0.001** |
| Group | 0.08 | -0.17 – 0.34 | 0.529 |
| Study Phase * Groupt | 0.07 | -0.16 – 0.30 | 0.535 |
|  |  |  |  |
| **Random Effects** |  |  |  |
| σ^2^ | 0.14 |  |  |
| τ_00_ _participantid_ | 0.21 |  |  |
| ICC | 0.61 |  |  |
| N _participantid_ | 82 |  |  |
| Observations | 163 |  |  |
| Marginal R^2^ / Conditional R^2^ | 0.105 / 0.649 |  |  |

4.2.4

| **Disturbing Dreams and Nightmares Severity Index (DDNSI) Total Score** | | | |
| --- | --- | --- | --- |
| *Predictors* | *Estimates* | *CI* | *p* |
| Intercept | 2.6 | 2.31 – 2.89 | **<0.001** |
| Sex | -0.01 | -0.17 – 0.15 | 0.914 |
| Age | 0 | -0.01 – 0.01 | 0.7 |
| Study Phase | -0.16 | -0.30 – -0.03 | **0.02** |
| Group | -0.1 | -0.27 – 0.08 | 0.27 |
| Study Phase * Groupt | 0.15 | -0.04 – 0.34 | 0.13 |
|  |  |  |  |
| **Random Effects** |  |  |  |
| σ^2^ | 0.09 |  |  |
| τ_00_ _participantid_ | 0.06 |  |  |
| ICC | 0.42 |  |  |
| N _participantid_ | 78 |  |  |
| Observations | 150 |  |  |
| Marginal R^2^ / Conditional R^2^ | 0.023 / 0.433 |  |  |

4.2.5

| **Epworth Sleepiness Scale (ESS) Total Score** | | | |
| --- | --- | --- | --- |
| *Predictors* | *Estimates* | *CI* | *p* |
| Intercept | 2.16 | 1.74 – 2.58 | **<0.001** |
| Sex | -0.19 | -0.42 – 0.04 | 0.107 |
| Age | 0 | -0.01 – 0.01 | 0.683 |
| Study Phase | -0.13 | -0.29 – 0.04 | **0.126** |
| Group | -0.03 | -0.28 – 0.21 | 0.787 |
| Study Phase * Groupt | 0.03 | -0.20 – 0.25 | 0.823 |
|  |  |  |  |
| **Random Effects** |  |  |  |
| σ^2^ | 0.13 |  |  |
| τ_00_ _participantid_ | 0.18 |  |  |
| ICC | 0.58 |  |  |
| N _participantid_ | 82 |  |  |
| Observations | 161 |  |  |
| Marginal R^2^ / Conditional R^2^ | 0.036 / 0.594 |  |  |

4.3 Fear Conditioning Results

4.3.1

|  | **Maximum SCR** | | | |  |  |
| --- | --- | --- | --- | --- | --- | --- |
|  | *Df* | *Sum Sq* | *Mean Sq* | *F Value* | *pr(>F)* | *σ2* |
| Age | 1 | 1.463 | 1.4627 | 3.09 | 0.0839 | 0.0500 |
| Sex | 1 | 0.44 | 0.4402 | 0.93 | 0.3388 | 0.0200 |
| Group | 1 | 0.562 | 0.5619 | 1.187 | 0.2803 | 0.0200 |
| Residuals | 60 | 28.404 | 0.4734 |  |  |  |

4.3.2

|  | **Differential “Conditionability” SCR (SCRd)** | | | |  |  |
| --- | --- | --- | --- | --- | --- | --- |
|  | *Df* | *Sum Sq* | *Mean Sq* | *F Value* | *pr(>F)* | *σ2* |
| Age | 1 | 0.009 | 0.00865 | 0.065 | 0.8 | 0.0011 |
| Sex | 1 | 0.001 | 0.00094 | 0.007 | 0.933 | 0.0001 |
| Group | 1 | 0 | 0.00002 | 0 | 0.989 | 0.0000 |
| Residuals | 60 | 8.001 | 0.13336 |  |  |  |

4.3.3

|  | **Extinction Learning Index (EXTidx)** | | | |  |  |
| --- | --- | --- | --- | --- | --- | --- |
|  | *Df* | *Sum Sq* | *Mean Sq* | *F Value* | *pr(>F)* | *σ2* |
| Age | 1 | 5054 | 5054 | 2.539 | 0.116 | 0.0400 |
| Sex | 1 | 3170 | 3170 | 1.593 | 0.212 | 0.0300 |
| Group | 1 | 4 | 4 | 0.002 | 0.966 | 0.0000 |
| Residuals | 60 | 119420 | 1990 |  |  |  |

4.3.4

|  | **Extinction Recall Index (ERI)** | | | |  |  |
| --- | --- | --- | --- | --- | --- | --- |
|  | *Df* | *Sum Sq* | *Mean Sq* | *F Value* | *pr(>F)* | *σ2* |
| Age | 1 | 0.062 | 0.0616 | 0.27 | 0.6052 | 0.0045 |
| Sex | 1 | 0.228 | 0.2277 | 0.998 | 0.3218 | 0.0200 |
| Group | 1 | 1.158 | 1.1581 | 5.077 | **0.0279** | 0.0800 |
| Residuals | 60 | 13.688 | 0.2281 |  |  |  |

4.3.5

|  | **Extinction Recall Magnitude (ERM)** | | | |  |  |
| --- | --- | --- | --- | --- | --- | --- |
|  | *Df* | *Sum Sq* | *Mean Sq* | *F Value* | *pr(>F)* | *σ2* |
| Age | 1 | 0.007 | 0.0066 | 0.047 | 0.8283 | 0.0008 |
| Sex | 1 | 0.263 | 0.2634 | 1.897 | 0.1735 | 0.0300 |
| Group | 1 | 0.602 | 0.6017 | 4.334 | **0.0416** | 0.0700 |
| Residuals | 60 | 8.331 | 0.1389 |  |  |  |

4.4 Heart Rate Variablitity Results

4.4.1

| **Root Mean Square of Successive Differences (RMSSD)** | | | |
| --- | --- | --- | --- |
| *Predictors* | *Estimates* | *CI* | *p* |
| Intercept | 3.69 | 3.21 – 4.17 | **<0.001** |
| Sex | -0.04 | -0.30 – 0.22 | 0.742 |
| Age | -0.02 | -0.03 – -0.01 | **0.006** |
| Study Phase | 0.08 | -0.14 – 0.30 | 0.475 |
| Group | 0.21 | -0.08 – 0.50 | 0.154 |
| Study Phase * Groupt | 0.03 | -0.29 – 0.34 | 0.863 |
|  |  |  |  |
| **Random Effects** |  |  |  |
| σ^2^ | 0.19 |  |  |
| τ_00_ _participantid_ | 0.14 |  |  |
| ICC | 0.41 |  |  |
| N _participantid_ | 60 |  |  |
| Observations | 120 |  |  |
| Marginal R^2^ / Conditional R^2^ | 0.113 / 0.476 |  |  |
